# Current Approaches to the Management of Patients with Endometrial Cancer

**DOI:** 10.3390/cancers14184500

**Published:** 2022-09-16

**Authors:** Emmanouil Kalampokas, Georgios Giannis, Theodoros Kalampokas, Angeliki-Astero Papathanasiou, Dimitra Mitsopoulou, Evangelia Tsironi, Olga Triantafyllidou, Mahalakshmi Gurumurthy, David E. Parkin, Mary Cairns, Nikolaos F. Vlahos

**Affiliations:** 1 Unit of Gynecologic Oncology, Second Department of Obstetrics and Gynecology, Aretaieion Hospital, Medical School, National and Kapodistrian University of Athens, 115 28 Athens, Greece; 2 Medical School, National and Kapodistrian University of Athens, 115 28 Athens, Greece; 3 Unit of Obstetrics and Gynecology, Second Department of Obstetrics and Gynecology, Aretaieion Hospital, Medical School, National and Kapodistrian University of Athens, 115 28 Athens, Greece; 4 Department of Gynaecological Oncology, Aberdeen Royal Infirmary, Aberdeen AB25 2ZN, UK; 5 Honorary Consultant Gynaecologist Oncologist, Aberdeen Royal Infirmary, Aberdeen AB25 2ZN, UK

**Keywords:** endometrial cancer, classification, management, lymphadenectomy, sentinel lymph node biopsy

## Abstract

**Simple Summary:**

New data, the development of new methods of treatment and management and the rising incidence of endometrial cancer require constant reviewing, in order to inform healthcare professionals about the current approaches to endometrial cancer. This review aims to present old, new and emerging perspectives in the management of endometrial cancer and evaluate the existing therapeutic strategies, by assessing the different surgical routes, the position of adjuvant therapies in the treatment of endometrial cancer and the implementation of SLNB. Upon reviewing literature data, it became clear that minimally invasive surgery is becoming gradually the preferred route of surgery, whereas SLNB is gaining more ground and could yield important information about the management needed in low-risk and high-risk endometrial cancer.

**Abstract:**

The incidence of endometrial cancer (EC) is rising and healthcare professionals need to be informed about the latest data on the constant developments in the field of its management. With particular interest in the classification and management of EC, we surveyed current literature, national and international data, and guidelines, as well as the latest studies to present the most recent data regarding the management of EC. It became evident that despite the consensus on low-risk EC, there are still controversies surrounding the management of high-risk EC, especially regarding the role of sentinel lymph node biopsy (SLNB). Our aim is to present the old and new perspectives in the management of EC, the different available surgical routes, the possible desire for fertility preservation, the role of adjuvant therapies and the focus on the advantages and the limitations of the implementation of SLNB in therapeutic strategies. It became evident throughout our search and based on literature data that minimally invasive surgery (MIS) leads to satisfying outcomes, thus becoming gradually the preferred route of surgery, while SLNB could provide essential information and guidance about the overall management needed in cases of both low-risk and high-risk EC.

## 1. Introduction

Endometrial cancer (EC) is the most common cancer of the female reproductive tract in developed countries, accounting for 3.4% of new cancer cases and for 4% of total cancer mortality among women in the US [1,2,3]. Its incidence varies among regions and it is continuously rising, along with its death rate, accelerating to 1.9% between 2008–2018 from 0.3% between 1997–2008, as the population is aging, the obesity rates are increasing and the prognosis of certain types of EC, mainly non-endometrioid EC, remains doubtful [3,4,5,6,7]. Wide-scale genome studies have contributed to a better understanding of carcinogenesis in uterine tumors and the integration of molecular biomarkers into international risk stratification systems. Additionally, innovative therapeutic strategies are being developed, such as immunotherapeutic agents that target selective molecules in DNA Polymerase Epsilon, catalytic subunit (POLE) mutated genes or in tumors with microsatellite instability (MSI). Staging is crucial and evincive for tumor management and prognosis, and it contains assessment of the primary tumor and the lymph nodes, and identification of distant metastases. Surgical resection remains the cornerstone of cure for EC, with minimally invasive surgery (MIS) offering multiple advantages. Advances are also presented in fertility-sparing options for younger patients diagnosed with EC, if desired. The best method of assessing lymph node status is still under investigation. Lymphadenectomy of pelvic and para-aortic lymph nodes has a role in the management of EC in some cases. However, in the surgical management of EC, sentinel lymph node biopsy (SLNB) allows mapping of the primary lymphatic pathway of the uterus, detecting metastases early, and minimizing mortality [8].

## 2. Classification of Endometrial Cancer

### 2.1. Histological Classification of Endometrial Cancer

EC arises from the endometrium, the inner mucosal layer of the uterine cavity [9]. In 2014, the World Health Organization established a histological classification system based on the morphology of the cells proliferating, consisting of six different histological types of EC: adenocarcinoma or endometrioid EC (EEC), clear cell EC, serous EC, mixed cell EC, carcinosarcoma and undifferentiated/dedifferentiated carcinoma. Histopathological evaluation that would define the tumor stage was believed to be an essential tool for EC risk stratification, further refining the therapeutic strategy [10].

The histological subtypes of EC present differences in incidence, prognosis, pathogenicity, and causality [3]. EECs comprise 75–80% of all EC cases, while serous and clear cell ECs are less common, accounting for 10% and 4% of newly diagnosed ECs, respectively [11,12]. The EC types also differ in histological features: EECs have diffuse glandular differentiation, that are squamous or secretory, appearing like normal endometrium [13]. There are several subtypes of EEC, such as secretory adenocarcinoma, villoglandular adenocarcinoma, and adenoacanthoma/acanthosquamous carcinoma [4]. Serous EC is characterized by papillary and/or glandular growth pattern [14] with marked cytological atypia, irregular luminal borders and usually develops from endometrial polyps or an atrophic endometrium [15]. Clear cell EC usually appears at an advanced stage at diagnosis, is characterized by tubulocystic, papillary or solid growth pattern, and presents a greater risk for recurrence and death [5,13,16].

In 1983, Bokhman [17] proposed the classification of EC based on different hormonal and metabolic mechanisms and introduced two clinicopathological types [17]. Type I EC, which is more common (~70–80%), corresponds to EEC and is usually associated with an expression for estrogen receptor (ER) [18]. Patients with Type I EC are usually obese and have multiple comorbidities, such as diabetes, hyperlipidemia, and metabolic syndrome, while infertility and anovulatory bleeding are also often present [1,19]. The majority of Type I EC cases are low-grade EECs, localized and confined to the uterus, with high overall survival (OS) rates [13]. Type II ECs are thought to be non-endometrioid, poorly differentiated, and do not express ER [1]. Approximately 65% of them are high-grade tumors with aggressive clinical presentation and a higher risk of metastasis and recurrence [20].

Later, it became evident that the pathogenesis of the two types of ECs Bokhman presented are not as distinct as primarily thought since the two tumor types are substantially heterogeneous and metabolic and endocrine signals between them overlap [20]. Moreover, it was proven that risk factors that were thought to be distinct for each type were actually associated with both types [21]. For example, ER expression, primarily thought to be a distinctive characteristic between Types II and I, is found in 95% of low-grade EEC/Type I but only in 15–50% of Grade 3 EEC/Type I, while more than 50% of serous carcinomas (Type II) actually have ER expression [22]. The Bokhman classification system currently has no practical utility [21].

### 2.2. FIGO Grading System

The International Federation of Obstetrics and Gynecology (FIGO) proposed a grading system for EC based on cellular architecture and the degree of glandular differentiation [3]. This system is only applicable to EECs or mucinous adenocarcinomas that have similar architecture and differentiation to EECs. The other types of ECs are high grade by definition. According to FIGO, EECs are divided into three categories: Grade 1 when they exhibit 95% or more of glandular squamous growth and up to 5% of solid growth pattern, Grade 2 when they contain 6–50% solid growth tissue, and Grade 3 when they have glandular differentiation in less than half (<50%) of cancer tissue and solid growth in greater than 50% of the tissue [23,24].

Since Grades 1 and 2 ECs share common epidemiological and molecular features, a new system called the “binary FIGO scheme” is now preferably used. In this scheme, Grades 1 and 2 tumors are designated “low-grade” and Grade 3 tumors are in the “high-grade” category [15].

However, the FIGO grading system has limitations and histomorphologic criteria are not adequate in successfully stratifying EC risk. The histological diagnosis based on histomorphological and immunochemical features is mostly delayed since tumors should first be surgically excised, especially high-grade ECs (Grade 3 EECs, serous, etc.). It is difficult to distinguish the different tumor types and classify them accurately. Even among skilled and experienced pathologists, one study found that in 38% of 131 Grade 3 EC cases, there was a reclassification of their histological grades [25,26].

Evidently, there is a need to develop a new, more representative grading system that will obtain faster and more biologically informative data for every tumor type.

### 2.3. Molecular Classification

In 2013, “The Cancer Genome Atlas” study collected data from wide-genome studies, proteomic analyses and MSI assays and performed an integrated genomic analysis assuming four molecular subgroups to further characterize and stratify endometrial tumors. This classification system is the most widely comprehensive and accepted [20,27].

Molecular classification may also reallocate a sizable portion of patients to a separate risk group, and algorithms are being developed to perform more targeted tests and thus reduce the number of tests required without impacting the risk classification [28].

The subgroups that occurred are presented in Table 1.

### 2.4. Pathogenesis

The pathogenesis of EC is influenced by certain gene mutations involved in molecular signaling pathways. Disruption in these pathways leads to inhibition of apoptosis, cell proliferation, telomere reverse transcription enhancement, or defects in DNA repair. EECs are prominently affected by alterations in AT-rich interaction domain 1A (ARID1A), phosphate and tensin homolog (PTEN), Kirsten rat sarcoma (KRAS) viral oncogene homolog, Catenin beta-1 (CTNNB1), and mismatch repair (MMR) molecular pathways. Alterations in tumor protein p53 (TP53), human epidermal growth factor 2, cyclin-dependent kinase inhibitr 2A (CDKN2A), Cyclin E1 (CCNE1), and F-box/WD repeat-containing protein 7 (FBXW7) genes are found in the majority of serous, high-grade endometrioid carcinomas and carcinosarcomas.

More specifically, the phosphatidylinositol 3-kinase (PI3K)–PTEN– serine/threonine kinase (AKT)– mammalian target of rapamycin (mTOR) pathway is altered in 80–95% of EECs. PTEN mutation results in the loss of protein function and increases the levels of phosphorylated AKT. Mutations of Phosphatidylinositol 3-Kinase Catalytic Subunit Alpha (PIK3CA) and Phosphatidylinositol 3-Kinase Regulatory Subunit 1 (PIK3R1) often occur synergistically with PTEN alterations. KRAS mutation occurs within the Rat sarcoma virus –Rapid accelerated fibrosarcoma –Mitogen-activated protein kinase/ERK kinase –Extracellular- signal- regulated kinase (ERK) pathway and affects 15–24% of EECs. In the canonical Wingless/int–β-catenin pathway, the main alteration occurs in the CTNNB1 gene, in which a gain-of-function mutation prevents the phosphorylation and ubiquitin-mediated proteasomal degradation of β-catenin. Despite the epigenetic silencing of the MutL protein homolog 1 (MLH1) gene that leads to MMR deficiency, frameshift mutations in the ataxia telangiectasia and Rad3-related protein, CCCTC-binding factor, Janus kinase 1, Ring finger protein 43 and ribosomal protein L22 genes have been reported in MSI(+) carcinomas.

Concerning non-endometrioid carcinomas, altered p53 expression is often accompanied by somatic mutations in Protein Phosphatase 2 Scaffold Subunit Aalpha, FBXW7, Speckle-type POZ protein, Chromodomain Helicase DNA Binding Protein 4 and TATA-Box Binding Protein Associated Factor 1 genes, while Erb-B2 Receptor Tyrosine Kinase 2, MYC proto-oncogene, bHLH transcription factor, CCNE1and CDKN2A are amplified or overexpressed [15].

### 2.5. Risk Factors

Epidemiological studies have revealed modifiable or non-modifiable factors with a positive or negative causal relationship with EC. They can be genetic, endocrine, immunological, epidemiological, demographic (such as age or ethnicity), or environmental [9,29].

Estrogen dominance comprises a principal driving factor for the development of endometrial cancer [4,30].

Unopposed estrogen effect on the endometrium results in excess cell proliferation, endometrial tissue hyperplasia, and malignancy. Obesity is one of the main endogenous sources for unopposed estrogen exposure and the single most important risk factor for EC, even when estrogen levels are normal [9,31,32].

Increased BMI results in increased levels of adipose tissue, in which the extraglandular conversion of androgens to estrogens occurs, mediated by aromatase [33]. Obesity increases insulin resistance and the circulatory levels of insulin and lowers the sex hormone-binding globin (SHBG) synthesis in the liver. Low SHBG levels lead to increased bioavailability of estradiol and testosterone, which induce cellular proliferation and inhibition of apoptosis in endometrial tissue [4].

Obese women tend to have metabolic syndrome and comorbidities such as diabetes mellitus and hypertension, and polycystic ovary syndrome, which contribute to EC development [33,34,35]. Recent data from Mendelian randomization protocols have indicated that hypercholesterolemia with high low-density lipoprotein cholesterol levels, though a common finding in metabolic syndrome, has been shown to decrease EC risk [34]. Insulin growth factor 1 (IGF-1) has a negative effect on EC, increasing the risk of its occurrence. IGF-1 effects this by mediating estrogen receptor expression when estradiol is lacking, resulting in alteration of concentration and bioavailability of estrogens in the body [4]. Exogenous exposure to estrogens includes prolonged hormone replacement therapy, which can increase the risk of EC development up to 20-fold [33]. The use of combined oral contraceptive pills, intrauterine devices, and tubal ligation act protectively on the endometrium [4]. Early onset of menarche and delayed menopause are related to increased EC development risk [35]. Nulliparity is an affirmed risk factor, especially if infertility is also present, while pregnancy and multiparity offer protection against EC. Previous breast malignancy may subsequently be followed by a second primary endometrial tumor. Very rarely does breast cancer metastasize to the uterus. Prior treatment with tamoxifen is also a well-established risk factor that doubles or triples the risk of EC [36].

Regarding modifiable risk factors, consistent physical activity and, paradoxically, smoking have been shown to reduce risk [4].

Non-modifiable factors, such as age have a linear correlation with endometrial carcinogenesis, as the majority of ECs (85%) are primarily diagnosed in post-menopausal women [35,37,38].

Racial disparities have also led to survival differences between black and white women, resulting in a higher prevalence of high-grade tumors in black women and in increased mortality rates (75%) [39] and they account for 58% of EC diagnoses among black women [40].

Genome-wide association studies have identified sporadic and hereditary genetic risk factors for EC. About 5% of EC cases are caused by genetic mutations. Well-studied syndromes that are correlated with EC are: Lynch syndrome, with autosomal dominant inheritance pattern and germ line mutations to MMR genes (MLH1, MutS Homolog 2 (MSH2), MutS Homolog 6 (MSH6) and postmeiotic segregation increased 2 (*S. cerevisiae*) (PMS2) [41]; Cowden syndrome, characterized by PTEN homolog mutations and development of multiple hamartomas, breast, colorectal, thyroid, kidney, and skin cancers; and polymerase proofreading associated polyposis, an autosomal dominant cancer predisposition syndrome attributed to germline mutations in the exonuclease domain of DNA Polymerase Delta 1, Catalytic Subunit or POLE genes [42].

### 2.6. Risk Stratification and Staging

Knowing the pathophysiological mechanisms and the etiological factors of endometrial tumors, it is possible to stratify risk to determine the optimal therapeutic management for every woman. In 2021, the European Society of Gynaecological Oncology (ESGO), the European Society for Radiotherapy and Oncology (ESTRO) and the European Society of Pathology (ESP) updated the existing staging guidelines [43,44] integrating FIGO staging, molecular classification, and grading, based on the best available multidisciplinary evidence and experience. Risk categories are as follows:

1. Low
2. Intermediate
3. High-intermediate
4. High and
5. Advanced/metastatic [1].

FIGO staging is assigned by examining myometrial depth of invasion, cervical involvement, nodal disease, and distant metastases, and they are presented in Table 1.

The “advanced metastatic” category, which is not mentioned in the Table, entails Stage III–IVA carcinomas with residual disease of any molecular subtype and Stage IVB tumors of any molecular status [45].

All the therapeutic decisions discussed below—surgical or medical treatment, fertility preservation, radicality of surgery, lymph node management, radiotherapy, etc.—are based on this risk classification system [44].

## 3. Management of Endometrial Cancer

### 3.1. Primary Management

Surgery remains the primary treatment option for EC. It usually includes total hysterectomy (TH), where the uterus and the cervix are removed, with bilateral salpingo–oophorectomy (BSO), where both fallopian tubes and ovaries are also removed [1,46,47]. Lymph node assessment is an incorporated part of the surgical management of EC and provides a strong predictor of survival, but the best method for this still raises controversy [48]. Surgical staging is essential, as it strongly determines prognosis and guides the decision for adjuvant treatment [1].

Generally, a meticulous abdominal and pelvic examination must be performed upon entering the peritoneal cavity. When possible, obvious extrauterine disease or suspicious lesions should be removed or biopsied [1]. It is suggested, though, that cytology of the peritoneal fluid should not be routinely performed, as positive results could be attributed to uterus handling. For some high-grade histological types of non-endometrioid EC, such as clear cell or serous carcinoma, and carcinosarcoma, omentectomy should be included in the surgical procedure, as visual assessment of the omentum appears to be insufficient [1,49,50]. When complete or optimal cytoreduction is attained, OS and progression-free survival (PFS) appear to be longer [51].

Oophorectomy is included in the standard procedure to exclude any ovarian metastasis or primary ovarian tumors, especially in patients with Lynch syndrome. However, ovarian preservation can be considered in young, premenopausal patients who have endometrioid type I EC, with approximately 50% endometrial invasion and no sign of extrauterine disease in preoperative diagnostic procedures [47]. Methods of fertility preservation, if desired, will be further discussed.

Radical hysterectomy, type II or III, is recommended in cases of clear cervical involvement [1,46].

Vaginal hysterectomy is also an option that might be used if the patient is not a suitable candidate for systematic surgery [46].

### 3.2. Routes of Surgery

The available routes of surgery are open surgery/laparotomy or MIS, which have emerged in recent years as the optimal methods and mainly include laparoscopic surgery (LS) and robot-assisted surgery (RS).

Combining the results of two of the largest randomized controlled trials, namely the Laparoscopy Compared with Laparotomy for Comprehensive Surgical Staging of Uterine Cancer: Gynecologic Oncology Group Study (LAP2) and the Laparoscopic Approach to Cancer of the Endometrium (LACE) trials, which compared laparotomy to laparoscopy, revealed multiple advantages of the latter [52,53]. LS is associated with a shorter hospital stay, enhanced recovery, and equal-to-laparotomy detection rates for overall disease at advanced stages. Fewer postoperative complications, such as blood loss, need for blood transfusion, wound complication, or need for ICU admission were also noted [51,54,55]. Furthermore, LS was shown to be more cost effective, especially in patients with higher BMI [54]. Despite longer operation times, LS was not accompanied by an increase in intraoperative injuries [55]. However, it is important to mention that the LAP2 trial enrolled patients with stage I to IIA EC, while the LACE trial included patients with stage I EC. These results concerning early-stage EC were confirmed in a recent Cochrane review [56]. Another study in 2020, showed that MIS was equally safe for Stage IIIC, without impairing survival and complete resection of disease was achieved [57]. More studies concerning high-grade ECs are needed.

An alternative effective type of MIS, RS has emerged and its use in EC is rising. Compared to both laparotomy and LS, RS resulted in even shorter hospital stays and fewer complications, namely, blood loss and blood transfusions [54]. Although RS is more expensive [1] and often associated with a longer duration of operation, a randomized trial found shorter operation times, and no conversions to open surgery [58]. Generally, RS is associated with fewer conversions to laparotomy, which mostly happen due to inadequate exposure [54]. The elderly, and patients with higher BMI, are also suitable candidates for RS [54,59]. However, recent studies have questioned the long-term outcomes of RS. More specifically, Argenta et al. [60] reported that in patients with stage I EC, RS is associated with poorer long-term outcomes, compared to the LS group, and led to poorer recurrence-free (Hazard Ratio-HR: 1.41; 95% CI: 1.12, 1.77), OS (HR: 1.39; 95% CI: 1.06, 1.83), and disease-specific survival (HR: 3.51; 95% CI: 2.19, 5.63). The study concludes that possibly, the significance of long-term effects has been underestimated due to positive short-term outcomes [60], as also highlighted in the British Gynaecological Cancer Society (BGCS) guidelines [47].

The benefits of MIS compared to open surgery have been established concerning both early and advanced EC stages as outlined in a retrospective cohort study in England published in 2020 [61]. The need for longer hospitalization periods with open surgery compared to MIS was confirmed (5.28 vs. 2.32 days), while the overall conversion rate for MIS was 6.6%. The only complication that was not significantly higher with open surgery was ureteric complications. Significantly higher overall 90-day mortality with open surgery (OR 0.34; 95% CI: 0.18–0.62; *p* = 0.0002) was also noted [61]. MIS has also been associated with reduced postoperative pain [54].

Currently, the BGCS suggests MIS for suitable patients [47], while ESGO, ESTRO, and ESP recommend MIS, even in patients with high-risk EC [44].

### 3.3. Determining Myometrial Invasion

The depth of myometrial invasion is essential for both staging and prognosis and it can be determined either pre-or intra-operatively. Magnetic resonance imaging (MRI) and transvaginal sonography (TVS) are the usual preoperative methods used, while intraoperative methods include intraoperative gross examination (IGE) and intraoperative frozen section (IFS). A 2016 meta-analysis, which mostly included studies with high-risk EC cases, in which deep invasion of myometrium is more common, showed the superiority of IFS compared to IGE in determining myometrial invasion. Pooled sensitivity and specificity for IFS were 85% and 97%, respectively, while for IGE they were 71% and 91%, respectively [62]. Another study compared the methods of MRI, TVS, IGE and IFS. It was observed that IFS had the highest sensitivity (90%), while IGE had the lowest sensitivity. For the preoperative methods, MRI had higher sensitivity than TVS, but both methods showed low positive predictive values [63]. IFS may be more time consuming, more expensive and demand the presence of a pathologist, which is not feasible in every center, but it prevails on determining tumor grade and its results are congruent with the results of histologic examination [64]. IGE, on the other hand, is quicker, cheaper, and simpler but vastly depends on the surgeon. It should be acknowledged that the tumor-invaded myometrium is not always macroscopically visible [62,63].

The implementation of sentinel lymph node (SLN) mapping will probably lower the need for IFS, but IFS can still be useful, especially in cases where the preoperative histopathological information is unclear or vague [64].

### 3.4. Fertility-Sparing Management

EC in younger patients might not be very common, but it is estimated that about 5% of cases involve patients under 40 years of age. In this group, it is important to consider the possible desire for fertility preservation. Fertility-sparing options, where the uterus and the ovaries are kept, can be considered if the following criteria are met: age younger than 40 years, endometrioid type EC, Stage I, with no evidence of myometrial invasion, and no evidence of metastatic disease, or lymph node involvement. Moreover, the expression of progesterone receptors on the endometrium is favorable and a strong predictor of remission, but it is not compulsory. Patients under 40 often meet the aforementioned criteria and they should be asked whether they desire to preserve their fertility so fertility-sparing options can be discussed. It should be clarified, though, that this is not the standard management of EC and existing risks and outcomes should be thoroughly discussed, accompanied by counseling [51,65].

Before any intervention, regular blood work, including Ca125, urine exams and endometrial biopsy should be conducted in the context of clinical staging [65]. Imaging, preferably with contrast–enhanced MRI, should also be performed to assess possible myometrial invasion and exclude any metastasis. Ovarian metastasis or synchronous ovarian cancer and the presence of Lynch syndrome should also be excluded. If the results are inconclusive or ambiguous, exploratory laparoscopy with peritoneal lavage, SLN biopsy, or biopsy of the ovaries could give more information. It is reported that in 5–30% of cases, these tests underestimate the tumor grade [51].

Hormonal methods are one of the options for fertility-sparing management. Oral progestin is used; and the most common regimens are medroxyprogesterone acetate and megestrol acetate. The response rates vary; in one study, 73% of cases responded to oral progestin, with a relapse rate of 36% [66], while in another study on tumors expressing estrogen and progesterone receptors, the response rate ranged from 26–89% when receptors were present, and from 8–17% when receptors were absent [65]. Alternatively, a levonorgestrel-releasing intrauterine device can be used. The use of gonadotropin-releasing hormone agonists (GnRHa) during chemotherapy in EC, to suppress ovarian function and limit ovarian damage is still under investigation [67].

Cryopreservation techniques are also available and they include embryo, oocyte, or ovarian tissue cryopreservation. The first two options require ovarian stimulation, while the last option does not, since cortical ovarian tissue can be obtained through laparoscopy [67]. Induction of ovulation seems not to be associated with higher relapse risk and in patients with estrogen-dependent tumors, there are strategies to maintain low estrogen levels during ovarian hyperstimulation [65].

Furthermore, in patients for radiotherapy, uterine transposition is performed under laparoscopy to keep the uterus, cervix, and ovaries in the upper abdomen, away from the area that will be radiated [68].

Once childbearing is complete, patients should undergo surgery with TH and bilateral salpingectomy [47,51].

### 3.5. Lymphadenectomy

Historically, surgical staging of EC has included complete pelvic and para-aortic lymphadenectomy [1], with the upper border at the left renal vein. As mentioned before, the nodal factor is essential in staging and provides important prognostic information [69], thus determining the need for adjuvant therapy [55], as well as the recurrence risk [70].

However, the role of lymphadenectomy in EC has raised controversy. The decision to perform a lymphadenectomy could depend on disease progression and tumor grade, as determined by preoperative imaging and biopsies [69].

It is known that the risk of lymph node involvement is correlated with the tumor grade, the depth of myometrial invasion and the high-risk histological types of EC. The results of early-stage EC from various studies tend to align. A recent Cochrane review, involving patients with Stage I EC who either underwent lymphadenectomy or did not, found no difference in overall and recurrence-free survival (RFS) between the two groups, concluding that routine use of lymphadenectomy is not recommended in early stages [69]. Considering higher-grade EC, however, given the higher risk of nodal involvement [48], many studies have shown a survival benefit when both pelvic and para-aortic lymphadenectomies are performed [1]. The need for para-aortic lymphadenectomy is also underlined by the fact that in approximately 8% of high-risk EC cases, while the pelvic nodes are negative, the para-aortic nodes are positive [51].

Nevertheless, full lymphadenectomy is associated with a number of adverse effects and high surgical morbidity [55]. Lymphedema, formation of lymphocysts, injury of blood vessels and pain and numbness from the lower abdomen to the genitalia and the inner thigh due to genitofemoral nerve damage are among the most common adverse effects. The incidence of lymphedema and lymphocysts may be even higher than reported, since many studies tend to focus on short-term effects. Deep vein thrombosis and pulmonary embolism could also occur postoperatively. Moreover, in obese patients, who represent a considerable percentage of patients with EC, the procedure presents multiple difficulties, mostly of a technical nature [69,70].

It is evident that randomized controlled clinical trials (RCTs) are needed to determine the role of lymphadenectomy in patients with higher-grade EC. In recent years, other less invasive methods have been in trial, such as the following:

1. Selective lymph node sampling;
2. Deciding whether to perform lymphadenectomy based on intrauterine risk factors mainly from IFS and;
3. SLN mapping.

The last method seems to offer the best results [55].

### 3.6. Adjuvant Radiotherapy

Radiotherapy is recognized among adjuvant modalities in EC standards of care and it includes pelvis external beam radiotherapy (PEBRT), whole pelvic radiotherapy, and vaginal brachytherapy (VBT). Risk stratification plays a key role in adjuvant radiotherapy (ART). Patients diagnosed with low-risk Stage I EC are not candidates for ART, as brachytherapy does not prevail over surgical management. While brachytherapy is recommended for intermediate-risk, high-risk and high-intermediate-risk EC, the presence of lymphovascular space invasion (LVSI) is of great importance regarding the latter EC group, in the presence of which external beam radiation therapy (EBRT) is also suggested. Several trials, such as the “A Study in the Treatment of Endometrial Cancer”/EN.5 (ASTEC/EN.5), Gynecologic Oncology Group (GOG)-99, Post Operative Radiation Therapy in Endometrial Carcinom (PORTEC)-1, and PORTEC-2, demonstrated no difference in OS, despite the effectiveness on pelvic and vaginal recurrences regarding early-stage intermediate-risk or high-risk EC. Although its effect on OS is limited, a pattern of concurrent chemoradiotherapy (CRT) is proposed for high-risk p53 + and high-risk Stages III C1 or C2 EC (PORTEC-3 protocol), where brachytherapy boost was indicated in cases of cervical involvement, LVSI or Grade 3 and Stage III EC [71,72,73,74].

Molecular profiles and biomarkers tend to upend the established therapeutic scenario in terms of ART. Interestingly, molecular classification of EC combined with clinicopathological factors of EC patients may suggest a novel risk profile as a determinant of ART in Stage I–II high-intermediate-risk EC (PORTEC-4a randomized trial) [71]. In a retrospective multicenter cohort study conducted by Reijnen et al. [75], proactive molecular risk classifier for endometrial cancer (ProMisE) MMR status was proposed as a predictive biomarker concerning ART response and, therefore, the effect of such treatment options on survival. The majority of the included cases concerned Stage II high-risk endometrioid EC [75]. Furthermore, Mohammadi et al. [76] proposed the use of the Radiosensitivity Index (RSI), a genomic signature, as a prediction model in pelvic recurrence, ART decisions and treatment escalation, including radiosensitizing agents [76].

### 3.7. Chemotherapy

Adjuvant chemotherapy remains the mainstay of high-risk EC treatment strategies. Carboplatin and paclitaxel are commonly used due to similar results in OS and PFS; they also appear to be less toxic. According to National Comprehensive Cancer Network (NCCN) guidelines, patients with clear cell or serous tumors of Stage IA characterized by myometrial invasion, Stage IB or Stage III could also be candidates for chemotherapy, with or without VBT. This could also be applied to Stages IB and II, Grade C EC. Stages III B and III C EC usually follow chemotherapy protocols in conjunction with radiotherapy, while adjuvant chemotherapy after cytoreductive surgery is suggested for resectable Stage IV disease.

Whether CRT is superior to adjuvant chemotherapy or radiotherapy alone remains controversial and current data lack prospective studies that compare adjuvant chemotherapy to adjuvant CRT. Radiation therapy (RT) could either be used before, after or in-between (sandwich fashion) chemotherapy.

The PORTEC-3 study showed that adjuvant chemotherapy (four cycles of carboplatin AwUC5 and paclitaxel 175 mg/m^2^) preceded by concomitant CRT (two cycles of cisplatin 50 mg/m^2^) improved five-year failure-free survival in patients with stage III EC, without increasing 5-year OS [72]. However, (GOG-249) study, a Phase III trial that included intermediate-high-risk stage II EC and Stage I to II clear cell or serous tumors, concluded that VBT followed by chemotherapy (three cycles of carboplatin AwUC6 and paclitaxel 175 mg/m^2^) (VBC/C) resulted in greater acute toxicity and nodal recurrences compared to the pelvic RT scheme. The differences between the two groups were minimal regarding late toxicity and 36-month OS and RFS [77]. On the contrary, Matei et al. [78] enrolled patients with Stage III or IVA (locally advanced) EC in a Phase III trial (GOG-258/NRG) and randomized them in a chemotherapy group and CRT (four cycles of carboplatin AwUC5-6 and paclitaxel 175 mg/m^2^) group. RFS was superior in the latter group, which was associated with a lower 5-year incidence of vaginal recurrence and pelvic and para-aortic lymph node recurrence, but with more distant metastases [78].

Results from the European Network of Gynaecological Oncological Trial-EN2- Danish Gynecological Cancer Group Phase III trial, in which postoperative chemotherapy (six cycles of paclitaxel-carboplatin) is compared with standard treatment (observation) in patients with node-negative Stage I and II intermediate-or high-risk EC, are expected. The primary outcome of the study is defined by OS [79].

Chemotherapy is also an effective tool against recurrent or metastatic EC, which usually does not respond to hormonal therapy. The GOG-209 trial compared the paclitaxel-doxorubicin-cisplatin regimen with carboplatin/paclitaxel (TC) in Stage III or IV EC. Both OS and PFS were similar between the two groups, while health-related quality of life was superior in the TC group [80]. GOG-286B, an ongoing Phase II/III study of Paclitaxel/Carboplatin/Metformin versus Paclitaxel/Carboplatin/Placebo may shed light on the beneficial effect of metformin in EC management concerning advanced and recurrent disease [81].

### 3.8. Immunotherapy

Recently, molecular classification has allowed targeted therapies to be developed. The angiogenesis pathway, the PI3K/Akt/mTOR pathway and glucose metabolism are under thorough investigation in studies, but currently there is no approved targeted therapy for this cancer beyond hormonal therapy [82].

A number of immune checkpoints and biomarkers are expressed in EC and immune system cells. Thus, their use in diagnostic tests, targeted therapeutic management, and predictive value could be promising. PD-1 and PD-L1 are recognized among these biomarkers and checkpoints, with the latter being mostly expressed in the POLE and MSI EC microenvironment. An algorithm presented by BGCS in 2022, suggests that MMR, p53 and Estrogen receptor (ER) immunochemistry should be performed on all EC, while POLE next generation sequencing should only be performed in cases with abnormal MMR and/or p53, stage I/II non endometrioid, grade 3 endometrioid, stage IA with no/focal LVSI, or endometrioid with either ER-negative, or stage IA with substantial LVSI, or stage IB/II [83]. Monoclonal antibody-based therapies, known as immune checkpoint blockade, have recently shown robust evidence in EC immunotherapy [84]. The “A Clinical Trial of Pembrolizumab (MK-3475) Evaluating Predictive Biomarkers in Subjects with Advanced Solid Tumors”, KEYNOTE-158, multicohort phase II study demonstrated the efficacy of pembrolizumab monotherapy, an anti-PD-1 agent, in MSI-H/dMMR advanced EC by denoting the antitumor activity and presenting a safe toxicity profile accompanied by a median PFS of 13.1 months [85]. Immunotherapy with a PD-1 inhibitor, combined with an antiangiogenic agent, when managing pretreated recurrent EC has been shown to be quite beneficial in patients’ survival. According to NCT03367741, a translational Phase II trial, cabozantinib-nivolumab combined therapy significantly improved PFS (5.3 vs. 1.9 months) [86]. Finally, a Phase III randomized placebo controlled trial aims to assess the addition of atezolizumab, an IgG1 PD-L1 inhibitor, to standard chemotherapy in advanced or recurrent EC; the results are expected in 2023 [87,88].

### 3.9. Sentinel Lymph Node Biopsy

SLN biopsy (SLNB) in EC is gaining ground in the staging of the disease and could be used as an alternative to selective lymphadenectomy (SLAD) and IFS. It is performed using tracer dyes (TD) and is oriented as the detection of at least one SLN in either or each hemipelvis. Blue dyes such as isosulfan blue, methylene blue and patent blue detected by colorimetry, indocyanine green (ICG) detected by near-infrared method, and Tc-99 m detected by radionuclide scanning are the most representative examples of TD-detector pairs. On the importance of preference, the near-infrared ICG method is usually chosen, as it is associated with higher detection rates and provides not only quick transcutaneous real-time visualization, but also low toxicity and cost. Its administration follows the pattern of “large volume and low concentration”. Detection rate is affected not only by the type of tracer, but also by the site of injection, LVSI, clinically enlarged lymph nodes, BMI, surgeon’s experience and RT history.

In uterine lymphatic draining systems, lymph nodes in the upper paracervical lymphovascular tissue are in favor of metastatic disease. The site of the TD injection could be *cervical, hysteroscopic myometrial/peritumoral and transabdominal subserosal/myometrial*. Despite the low risk of isolated para-aortic metastasis, a higher detection rate of para-aortic SLN mapping is offered by hysteroscopic injection. Nevertheless, cervical injection is considered the safest and easiest-performed method.

Positive SLN mapping includes macrometastases, micormetastases and isolated tumor cells. Hematoxylin-eosin stain is used for SLN pathological analysis. In negative results, ultrastaging is recommended, which consists of deep serial sections and cytokeratin immunohistochemical stain [8,55].

The most commonly used surgical algorithm for SLN mapping was described by Persson et al. in 2017. After the cervical injection, ICG display is bilaterally evaluated in the upper and lower paracervical pathway. If all four pathways are visible, the presacral avascular plane is opened in order to identify and remove SLNs along the lower paracervical pathway bilaterally. The next step is to dissect the paravesical and pararectal planes to identify and remove SLNs along the upper paracervical pathway. Lastly, the upper parametrium is removed and, along with the SLNs, is checked with ultrastaging and immunochemistry. If all pathways are not visible thoroughly, exploration and reinjection are the only available options before full lymph node dissection is required [89].

SLNB was considered experimental at the ESMO/ESGO/ESTRO consensus conference on EC in 2016 [90]. However, in 2021, the ESGO/ESTRO/ESP guidelines recommended SLNB for surgical staging in patients with low-risk or intermediate-risk EC, while systematic lymphadenectomy was not recommended in this group. In patients with high-intermediate-risk or high-risk disease, SLNB is considered an acceptable alternative to systematic lymphadenectomy [44].

According to Stewart et al. [8], surgical staging of both low-and high-risk EC through the SLN algorithm reduced operative time and the use of IFS. However, the SLN algorithm was not superior in terms of hospital charges or intraoperative and postoperative complications [8].

The prospective “A comparison of sentinel lymph node biopsy to lymphadenectomy for endometrial cancer staging” (FIRES) trial demonstrated SLN mapping using ICG as a technique with high sensitivity and negative predictive value in the detection of nodal metastases, enough to succeed staging through lymphadenectomy. The FIRES trial interpreted that SLN mapping with ICG has high diagnostic accuracy in detecting EC metastases and can therefore be a safe alternative to lymphadenectomy, with the benefit of exposing fewer patients to the morbidity of a complete lymphadenectomy. Specifically, the SLN technique had a sensitivity of 97.2% (95% CI: 85.0–100) and a negative predictive value of 99.6% (95% CI: 97.9–100), as 257 of the 258 patients had truly negative non-sentinel lymph nodes. Furthermore, SLN mapping proved to be superior to traditional complete lymphadenectomy, as pathologically identified SLNs were significantly more likely to contain metastatic disease compared to non-SLN, thus, pathologists were required to ultra-stage less, but more crucial, nodes [70].

Moreover, the distribution and typical position of SLNs in high-risk EC were investigated in the FIRES trial with SLN-ICG identifying SLNs in the obturator area, left and right, in approximately 60% of patients, in the external iliac area, bilaterally, in 80% of patients, in the presacral area in half the patients, and only 13% of patients had an SLN identified in the common iliac area. Metastatic SLNs were found mostly in the obturator area (25% of node-positive patients left/36% right) and in the external iliac area (41% left/25% right) [89].

A meta-analysis by Bogani et al. in 2019 [91] showed that SLN mapping was not only superior to lymphadenectomy, but also, in combination with pathological ultrastaging, it achieves a higher detection rate of nodal disease in comparison to lymphadenectomy and an accurate detection of positive nodes, even in high-risk EC. Low-risk patients who underwent SLN mapping had a higher detection rate of positive pelvic nodes (OR: 3.12 (95% CI: 1.32–7.39) and a comparable detection rate of paraortic nodes (OR: 1.38 (95% CI: 0.39–4.83)). This outcome was also observed in the intermediate and high-risk EC groups, with a higher detection rate in pelvic nodes (OR: 2.04 (95% CI: 1.19–3.48), and a similar detection rate between groups for positive para-aortic nodes. These data additionally support the effectiveness of SLN mapping in terms of oncologic outcome, as no statistical difference in RFS was observed between groups (OR: 0.90 (95% CI: 0.58–1.38) [91].

In high-risk EC, the “Pelvic Sentinel lymph node detection in High-Risk Endometrial Cancer” (SHREC) trial showed that pelvic SLN detection had a 100% sensitivity and a 100% negative predictive value with no adverse events during the SLN procedure. The researchers, however, noted that reinjection of tracer was crucial for the outcome, as bilateral mapping rate increased from 82%, prior to reinjection, to 95% after reinjection, thus suggesting consideration of reinjection of the tracer when mapping not being satisfactory [89].

In 2020, a meta-analysis by Ji et al. [92] assessing SLN mapping in high-risk EC concluded that SLN mapping has a high detection rate and diagnostic accuracy in high-risk EC, comprising a viable alternative to lymphadenectomy. More specifically, a pool detection rate of 87.8% (95% CI: 85.1–90.5%) was observed in 514 patients in seven different studies, with a pooled sensitivity of 87% (95% CI: 79–92%), a pooled specificity of 98% (95% CI: 96–99%) and a negative predictive value of 97.7% (95% CI: 96.4–99.1%) [92]. This conclusion is also supported by a study performed by Cusimano et al. [93] in 2021, which showed that 96% of patients with lymph nodes were correctly identified and 99% of patients with negative SLN had node-negative disease [93].

SLN detection rates are not observed to be associated with histology, average patient BMI, tumor grade, or surgical approach. Furthermore, no difference was found in PFS between patients who were treated with SLN mapping and primary lymphadenectomy [94].

It is evident that more data are emerging regarding SLNB in high-risk EC, as more studies are investigating its detection rate and diagnostic accuracy compared to systematic lymphadenectomy (see Table 2).

## 4. Discussion

Surgical staging is most important in the management of EC, and MIS is a suitable alternative to open surgery. Controversy surrounds the best method for the assessment of lymph nodes, an integral part of staging. With the latest guidelines by ESGO/ESTRO/ESP there seems to be a consensus concerning low-risk EC, and SLNB is the recommended treatment. High-risk EC is the main discussion topic, with many researchers focusing on determining the best available route. Complete pelvic and para-aortic lymphadenectomy are still the standard of care for high-risk EC, although the adverse effects and high surgical morbidity have led many trials to research the safety of SLNB in this population. More specifically, the FIRES [70] and SHREC [89] trials showed that SLNB is a safe alternative to lymphadenectomy, and the meta-analyses followed by Bogani et al. in 2019 [91] and Ji et al. in 2020 [92] confirmed the high detection rate and diagnostic accuracy of SLNB.

Another reason for the shift in the management of EC is the newly established molecular classification, as it has altered prognosis and decision-making regarding adjuvant treatment, not only changing the protocols of chemotherapy and radiotherapy but also introducing immunotherapy as a viable option. ART depends on risk stratification and low-risk stage I EC patients are not suitable candidates. Molecular profiles and biomarkers tend to alter the established management of EC, with a new prediction model (the RSI) proposed to guide ART decisions. Adjuvant chemotherapy remains the cornerstone of high-risk EC treatment strategies; it remains to be demonstrated whether CRT is superior to adjuvant chemotherapy alone, as there is a lack of prospective studies on this. Finally, anti-PD-1 agents, alone or combined with antiangiogenic agents, have yielded promising results for advanced or pretreated recurrent EC. More trials aiming to assess more targeted therapies for the management of EC are ongoing, with expected results as soon as 2023.

One point of interest in the future management of EC is fertility-sparing management whenever this is desired. Fertility-sparing options are present, if certain criteria are met, with many methods that make fertility preservation easier and safer. The advancement of hormonal methods and cryopreservation techniques, such as ovarian tissue cryopreservation, has brought more potential for the preservation of fertility. However, after childbearing is complete, surgery is inevitable.

From the findings of this paper, it is evident that there is a potent research field in every aspect of the future management of EC.

## 5. Conclusions

Surgery constitutes the cornerstone of the management of EC, with minimally invasive surgery gaining ground over open surgery. Adjuvant and targeted treatment strategies do not mitigate surgical management, but are essential assets in managing EC and ameliorating its overall prognosis. While the scientific community seems to be in agreement for the management of low-risk EC, the treatment strategies for high-risk EC are still controversial. There is an ongoing discussion concerning the preferred method for the assessment of lymph nodes, which is an integral part of surgery. Lymphadenectomy can still be performed, presenting multiple advantages, but sentinel lymph node biopsy emerges as an alternative even in high-risk EC. The increased incidence of EC makes the need for holistic management based on up-to-date data imperative, so as to improve and facilitate the lives of people affected by EC. Moreover, the fact that EC is also found in younger patients calls for healthcare professionals to be informed about fertility sparing options, should this be desired by the patients. New data emerge constantly and new arguments will undoubtedly surface. Ongoing clinical trials will empower current EC guidelines and will optimize the available methods for the management of EC.

## 6. Future Directions

More studies need to be performed to have a better understanding of the molecular classification and, therefore, of the risk stratification of EC. Furthermore, the value of SLNM in high-risk EC should be more thoroughly investigated so that more precise guidelines regarding the management and treatment of high-risk EC can be published, and lastly, fertility sparing options should be more entailed in the management of EC in younger women, with more studies being conducted relative to the issue.

## Figures and Tables

**Table 1:** Molecular classification of Endometrial Cancer and correlation with previous classification systems.

TCGA Subgroup	Mutated Genes	Genetic Abberation	Surrogate Marker	Prevalence	Histology	FIGO Grade	Stage	Risk Group	Recurrence Status	Prognosis
**Hypermutated MSI/MSI-H/MMRd**	MLH1, MSH2, MSH6, PMS2	Microsatellite instability, somatic or germline mutations in MMR genes and epigenetic changes (i.e., MSH1 silencing)	MSH6, PMS2 IHC expression	24.7% of G1–2 tumors	EEC	Low (G1–2)	IA	Low risk	LVSI (−) or focal	Variable
							IB	Intermediate		
						High (G3)	IA			
							IB	High-intermediate	Regardless of LVSI status	
				39.7% of G3 tumors		Regardless of the grade	I		Substanial LVSI	
							II		(−)	
						High	III–IVA	High risk	No RD	
					Non-EEC *		I–IVA		MI, no RD	
**Copy-number-low (CNL)/non specific molecular profile (NSMP)**	TP53 wild type	Low number of mutations, microsatellite stability	Normal p53 IHC expression	63.5% of G1–2 tumors	EEC	Low	IA	Low risk	LVSI (−) or focal	Variable
							IB	Intermediate		
						High	IA			
				28% of G3 tumors			IB	High-intermediate	Regardless of LVSI status	
						Regardless of the grade	I		Substanial LVSI	
							II		(−)	
						High	III–IVA	High risk	No RD	
					Non-EEC *		I–IVA		MI, no RD	
**Copy-number-high (CNH)/p53abn**	TP53	TP53 somatic mutation (91% of cases)	Aberrant p53 IHC expression or aneuploidy with simultaneous testing to exclude MSI-H or POLE	4.7% of G1–2 tumors	Non-EEC *	N/A	IA	Intermediate	Without MI	Unfavorable
				25% of G3 EEC	EEC or non-EEC *		I–IVA	High	MI, no RD	
**POLE ultramutated (POLEmut)**	POLE	Somatic mutation of POLE, TP53 mutation in 35% of cases	Exonuclease domain POLE gene/molecular analysis	6.2% of G1–2 tumors	EEC or non-EEC *	Low (G1–2)	I–II	Low	No RD	Excellent
				12.1% of G3 tumors		High (G3)				

* Non-endometrioid: clear cell, serous, undifferentiated, carcinosarcoma, mixed; LVSI = LymphoVascular Space Invasion, RD = residual disease, MI = myometrial invasion, EEC = endometrioid carcinoma; IHC = immunohistochemical, N/A = not applicable.

**Table 2:** Studies involving the role of SLNB in high-risk EC and their conclusion.

Study	Reference Standard	Conclusion
Naoura, 2015 [95]	Systematic bilateral pelvic lymphadenectomy +/− para-aortic lymphadenectomy	High-risk EC patients had a higher false negative rate, meaning its use is still doubtful in this population.
Baiocchi, 2016 [96]	Systematic pelvic +/- para-aortic lymphadenectomy	SLN-mapping + ultra-staging has a higher detection rate of node metastases. Para-aortic lymphadenectomy is not necessary for patients with negative SLN mapping.
Paley, 2016 [97]	Bilateral pelvic and periaortic lymphadenectomy	SLN-ICG is feasible and has high detection rates. Low false negative rates are promising and if confirmed in larger studies, SLN mapping could alter the surgical management of patients with EC.
Ehrisman, 2017 [98]	Complete pelvic lymphadenectomy	High-risk EC has a slightly lower detection rate when using the SLN mapping method, compared to lower risk cancers. However, using an SLN algorithm raises the Negative Predictive Value of SLN mapping alone from 92% to 100%.
Soliman, 2017 [99]	Full pelvic and para-aortic lymphadenectomy up to the renal vessels	SLN biopsy alone accurately identified 95% of patients with positive lymph nodes. Combined with side-specific lymph node dissection SLN biopsy had a false negative rate of 4.3% and a false negative predictive value of 1.4%, thus supporting the use of SLN mapping in high-risk EC, along with a side-specific lymphadenectomy algorithm if an SLN cannot be obtained.
Touhami, 2017 [100]	Complete pelvic lymphadenectomy ± para-aortic lymphadenectomy	SLN mapping using cervical injection in high-risk EC has high sensitivity and high negative predictive value, with only one false negative case occurring. SLN mapping, as a result, seems to be a suitable choice in this specific population.
Papadia, 2018 [101]	Full pelvic and para- aortic lymphadenectomy up to the renal vessels	NIR-ICG SLN mapping is a safe alternative to systematic lymphadenectomy in women with poorly differentiated EC.
Rajanbabu, 2018 [102]	Bilateral pelvic and paraaortic lymphadenectomy where SLN’s were not detected	SLN mapping surgical algorithm yielded a detection rate of 100% with no false negative cases in various EC risk-groups.
Ruiz, 2018 [103]	Pelvic and para-aortic lymphadenectomy	SLN had detection rates of 89.19% in the pelvic area and 59.46% in the para-aortic area, with an overall detection rate was 92.79%. As a result, SLN biopsy is an efficient compromise between systematic lymphadenectomy and no dissection.
Togami, 2018 [104]	Pelvic lymphadenectomy with or without para- aortic lymphadenectomy	SLN biopsy could help avoid, if not necessary, systematic lymphadenectomy and adverse effects, though the use of it in high-risk patients must be decided after careful thought.
Backes, 2019 [105]	Pelvic and para-aortic lymphadenectomy at the surgeon’s discretion	SLN mapping is feasible and a safe alternative for complete lymph node dissection
Wang, 2019 [106]	Pelvic with or without paraaortic lymphadenectomy	SLN mapping was successful in 86.7% of patients, with a false negative rate of 11.8% and a negative predictive value of 97.3%. SLN biopsy could be used to diagnose high-risk EC.
Ye, 2019 [107]	Complete bilateral lymphadenectomy ± paraaortic lymphadenectomy to the inferior mesenteric artery	SLN-ICG has a low sensitivity and a high false negative rate in high-risk EC and therefore is unacceptable in clinical practice.
Cusimano, 2021 [93]	Pelvic lymphadenectomy +/− para-aortic lymphadenectomy	SLN biopsy had allowable diagnostic accuracy and improved the detection of node-positive cases compared with lymphadenectomy in women with high-risk EC, thus supporting its viability as a method of surgical staging of high-risk EC.
Bogani, 2021 [108]	SLNM plus back-up lymphadenectomy	Back-up lymphadenectomy did not improve disease-free and overall survival in high-risk EC.
